# A chromosome-level genome assembly of the mountain lion, *Puma concolor*

**DOI:** 10.1093/jhered/esae063

**Published:** 2024-11-07

**Authors:** Megan A Supple, Merly Escalona, Nicolas Alexandre, Michael R Buchalski, Seth P D Riley, Justin A Dellinger, T Winston Vickers, Ruta Sahasrabudhe, Oanh Nguyen, Colin W Fairbairn, William E Seligmann, Christopher C Wilmers, Beth Shapiro

**Affiliations:** Department of Ecology and Evolutionary Biology, University of California, Santa Cruz, CA, United States; Howard Hughes Medical Institute, University of California, Santa Cruz, CA, United States; Department of Biomolecular Engineering, University of California, Santa Cruz, CA, United States; Department of Ecology and Evolutionary Biology, University of California, Santa Cruz, CA, United States; Howard Hughes Medical Institute, University of California, Santa Cruz, CA, United States; Wildlife Genetics Research Unit, Wildlife Health Laboratory, California Department of Fish and Wildlife, Sacramento, CA, United States; Santa Monica Mountains National Recreation Area, National Park Service, Thousand Oaks, CA, United States; Large Carnivore Section, Wyoming Game and Fish Department, Lander, WY, United States; Karen C. Drayer Wildlife Health Center, University of California, Davis, CA, United States; DNA Technologies and Expression Analysis Core Laboratory, Genome Center, University of California, Davis, CA, United States; DNA Technologies and Expression Analysis Core Laboratory, Genome Center, University of California, Davis, CA, United States; Department of Ecology and Evolutionary Biology, University of California, Santa Cruz, CA, United States; Department of Ecology and Evolutionary Biology, University of California, Santa Cruz, CA, United States; Environmental Studies Department, University of California, Santa Cruz, CA, USA; Department of Ecology and Evolutionary Biology, University of California, Santa Cruz, CA, United States; Howard Hughes Medical Institute, University of California, Santa Cruz, CA, United States

**Keywords:** California Conservation Genomics Project, conservation genomics, felidae, wildlife management

## Abstract

Mountain lions, *Puma concolor*, are widespread and adaptable carnivores. However, due to their large home ranges and long distance dispersals, they are strongly impacted by habitat fragmentation, which results in small and isolated populations. Genomic analyses play an important role in understanding and predicting the impacts of increased isolation of populations, such as decreased genetic diversity and increased levels of inbreeding. Here we report a high-quality, chromosome-level reference genome of *P. concolor* that was generated as part of the California Conservation Genomics Project. The primary assembly has a total length of 2.5 Gb contained in 258 scaffolds, a contig N50 of 42.3 Mb, a scaffold N50 of 149.8 Mb, and a BUSCO completeness score of 95%. This *P. concolor* genome assembly will provide an important resource for genomic analyses that aid decision-makers in managing the species in fragmented landscapes.

## Introduction

Mountain lions (*Puma concolor*, also known as pumas, cougars, or panthers) are a widespread species and a top predator in a variety of ecosystems across the Americas (Nowell and Jackson 1996). The modern lineage in North America was likely founded through colonization from South America, resulting in lower genetic diversity (Culver et al. 2000; Caragiulo et al. 2013; Saremi et al. 2019). Widespread persecution and subsequent local extirpation resulted in significant range contractions within North America (Kellert et al. 1996), further reducing genetic diversity for some populations. This is exemplified by the Florida panther population, which is a small, isolated population that had very low genetic diversity and suffered from phenotypic defects associated with inbreeding (Roelke et al. 1993). This population was genetically rescued by translocating a small number of Texas pumas into the population, which increased genetic diversity, decreased inbreeding, and ultimately increased fitness (Johnson et al. 2010; Onorato et al. 2024). While the International Union for Conservation of Nature (IUCN) Red List indicates the global mountain lion population is decreasing, the species is classified as Least Concern (Nielsen et al. 2015). In the United States, only the Florida populations are listed as endangered under the US Endangered Species Act (U.S. Fish and Wildlife Service 2024).

Some mountain lion populations in California are suffering from similar reduced genetic diversity and inbreeding depression as a result of habitat loss and fragmentation, with negative consequences for population viability (Ernest et al. 2014; Riley et al. 2014; Benson et al. 2016, 2019; Huffmeyer et al. 2022). The fragmented populations along the central and southern California coast are currently candidates for listing under the California Endangered Species Act due to low heterozygosity and small effective population sizes (Center for Biological Diversity and the Mountain Lion Foundation 2019; Gustafson et al. 2019; Dellinger et al. 2020). Given concerns about the viability of small, isolated populations, improved genomic tools that can inform management actions are critical.

Genomic resources, including high-quality genome assemblies, enable both empirical assessments of genetic diversity and simulations of population viability. Combining reference genomes and population-level whole-genome resequencing allows for the high-resolution assessment of deleterious mutational load and the extent of autozygosity due to close inbreeding. These estimates can be used to identify populations with reduced viability and to simulate the genomic consequences of different management scenarios (Benson et al. 2016, 2019). These population genomic analyses can provide managers with important information for making decisions about populations of conservation concern, such as the central and southern California coast mountain lion populations.

High-quality genome assemblies will also enable comparative genomic analyses across felid species. Felidae is a diverse family of species that occupy a wide variety of ecosystems. A comparative genomics study of five felid species revealed insights into genomic evolution and adaptation of this family (Bredemeyer et al. 2023). There are publicly available, chromosome-level assemblies for more than a dozen felid species, which will enable additional insights into the genomic mechanisms underlying adaptations in this diverse family. The current mountain lion RefSeq assembly in the National Center for Biotechnology Information (NCBI) database is a scaffold-level assembly, with the 18 autosomal chromosomes largely represented by 26 scaffolds (GCF_003327715.1; Saremi et al. 2019). New sequencing technologies and assembly methods have increased the contiguity of assemblies, which will improve inferences that are based on linked markers.

Here we report a high-quality, chromosome-level genome assembly generated from a California mountain lion as part of the California Conservation Genomics Project (CCGP; Shaffer et al. 2022). This genome assembly will be a valuable resource for management of the mountain lions across California and the rest of the Americas.

## Methods

### Biological materials

**Fig. 1.**
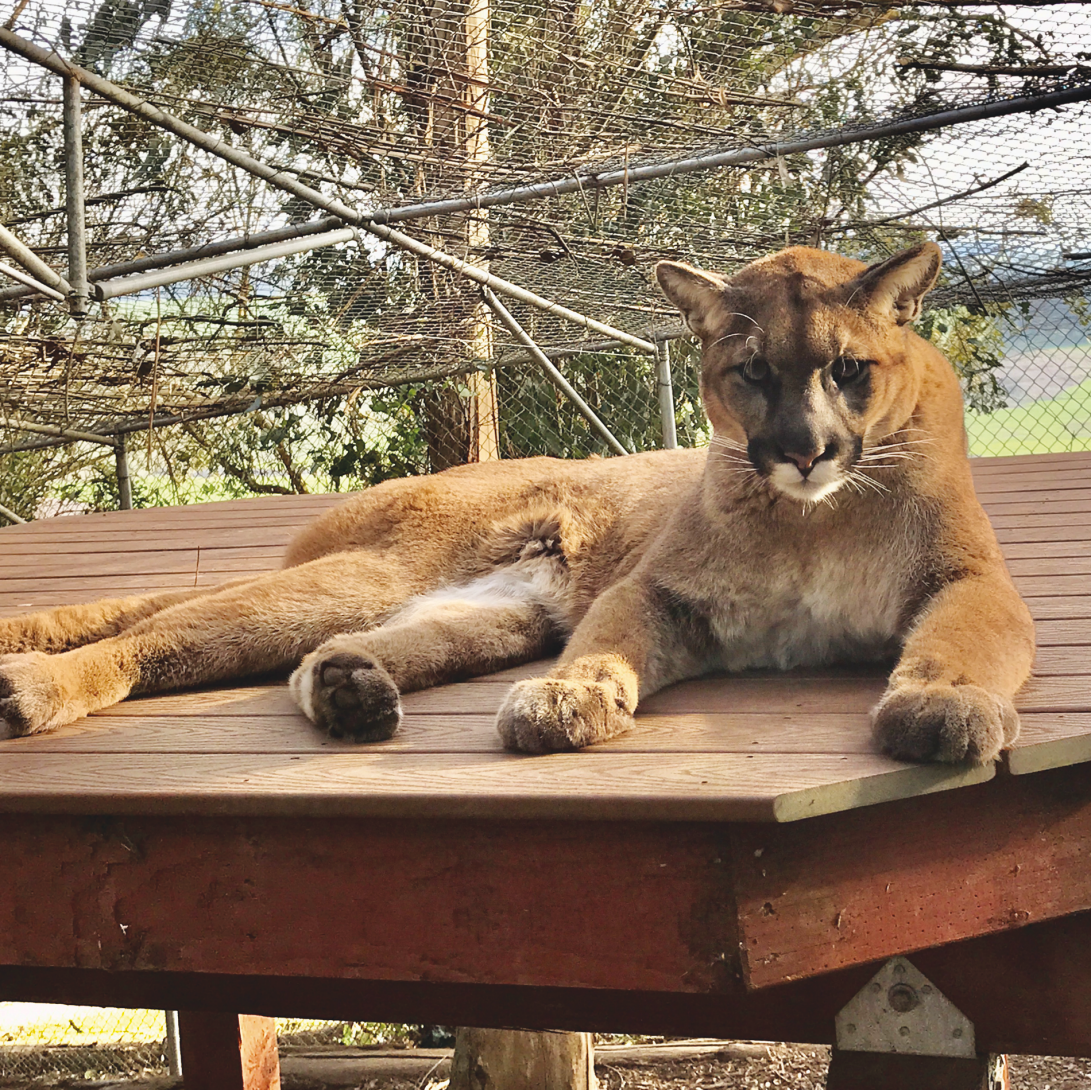
Charlie, the captive mountain lion who provided a blood sample for the genome assembly. Photo courtesy of Sonoma County Wildlife Rescue.

We collected a blood sample from an adult male mountain lion (Charlie, L17-30, Fig. 1). The mountain lion was rescued from the Thomas Fire near Santa Paula, CA, as a juvenile (~5 months old) in December 2017. Unable to be released back into the wild, in February 2018 he was transferred to Sonoma County Wildlife Rescue. In December 2020, a veterinarian drew the blood sample during a routine exam. The blood sample was collected into vacutainer tubes containing ethylenediaminetetraacetic acid (EDTA), transported on ice, and then transferred to cryovials and flash frozen in liquid nitrogen.

### Nucleic acid library preparation and sequencing

At the UC Davis Genome Center, we isolated high-molecular weight (HMW) genomic DNA (gDNA) from the whole blood sample. We added 6 ml of RBC lysis solution (Qiagen; Germany; Cat # 158445) to 2 ml of whole blood and incubated the reaction at room temperature for 5 min. We centrifuged the sample at 2000 × *g* for 5 min to pellet white blood cells. We discarded supernatant and added 2 ml of lysis buffer containing 100 mM NaCl, 10 mM Tris-HCl pH 8.0, 25 mM EDTA, 0.5% (w/v) sodium dodecyl sulfate (SDS), and 100 µg/ml Proteinase K to the pellet. We incubated the reaction at room temperature overnight. We treated the lysate with 20 µg/ml RNase A at 37 °C for 30 min and then cleaned with equal volumes of phenol/chloroform using phase lock gels (Quantabio; Beverly, MA; Cat # 2302830). We precipitated the DNA by adding 0.4× volume of 5 M ammonium acetate and 3× volume of ice-cold ethanol. We washed the DNA pellet twice with 70% ethanol and resuspended in an elution buffer (10 mM Tris pH 8.0). We assessed the purity of the gDNA using a NanoDrop ND-1000 Spectrophotometer (Thermo Fisher Scientific; Wlatham, MA) and observed a 260/280 ratio of 1.83 and 260/230 ratio of 2.38. We quantified the DNA yield at 25 µg with a Qubit 2.0 Fluorometer (Thermo Fisher Scientific). We verified the integrity of the HMW gDNA on a Femto pulse system (Agilent Technologies; Santa Clara, CA), confirming that 85% of the DNA was in fragments over 100 kb.

At the UC Davis Genome Center, we constructed a HiFi SMRTbell library using the SMRTbell Express Template Prep Kit v2.0 (Pacific Biosciences [PacBio]; Menlo Park, CA; Cat. #100-938-900) according to the manufacturer’s instructions. We sheared HMW gDNA to a target DNA size distribution of 15 to 20 kb using Diagenode’s Megaruptor 3 system (Diagenode; Belgium; Cat. B06010003) and then concentrated the sheared DNA using 0.45× of AMPure PB beads (PacBio; Cat. #100-265-900). We then processed the DNA through a series of enzymatic reactions: removal of single-strand overhangs at 37 °C for 15 min, DNA damage repair at 37 °C for 30 min, end repair and A-tailing at 20 °C for 10 min and 65 °C for 30 min, ligation of overhang adapters v3 at 20 °C for 60 min followed by 65 °C for 10 min to inactivate the ligase, and nuclease treatment at 37 °C for 1 h. We then purified and concentrated the SMRTbell library with 0.45× Ampure PB beads for size selection using the BluePippin/PippinHT system (Sage Science; Beverly, MA; Cat #BLF7510/HPE7510) to collect fragments greater than 9 kb, with a resulting average fragment size of 22 kb. We sequenced the HiFi SMRTbell library at UC Davis DNA Technologies Core using four 8M SMRT cells (PacBio; Cat #101-389-001), Sequel II sequencing chemistry 2.0, and 30-h movies for each cell on PacBio Sequel II and IIe sequencers.

At the UC Santa Cruz Paleogenomics Laboratory, we prepared an Omni-C library from whole blood using a Dovetail Omni-C Kit (Dovetail Genomics; Scotts Valley, CA) according to the manufacturer’s protocol with slight modifications. Briefly, we cross-linked the chromatin in the nucleus, digested the chromatin with DNase I, repaired chromatin ends and ligated a biotinylated bridge adapter to the repaired ends, reversed the cross-links, and purified the DNA. We treated purified DNA to remove biotin that was not internal to ligated fragments. We generated a short-read sequencing library using an NEB Ultra II DNA Library Prep Kit (New England Biolabs; Ipswich, MA) with an Illumina compatible y-adaptor. We captured biotin-containing fragments using streptavidin beads. We split the postcapture product into two replicates prior to polymerase chain reaction enrichment to preserve library complexity, with each replicate receiving a unique dual index. We sequenced the library at the UC Berkeley Vincent J. Coates Genomics Sequencing Laboratory (Berkeley, CA) on the Illumina NovaSeq 6000 platform (Illumina; San Diego, CA) with an S4 flow cell targeting 250 million 2 × 150-bp read pairs.

### Nuclear genome assembly

We assembled the genome of a male *P. concolor* individual following the CCGP assembly pipeline version 5.0 (https://github.com/ccgproject/ccgp_assembly). Table 1 lists the software and nondefault parameters used in the assembly process. First, we removed remnant adapter sequences from the PacBio HiFi dataset using HiFiAdapterFilt (Sim et al. 2022). We then generated an initial phased diploid assembly using HiFiasm (Cheng et al. 2022) in HiC mode using the filtered PacBio HiFi reads and the Omni-C short reads, a process that generates two assemblies, one per haplotype. We then aligned the Omni-C data to both assemblies following the Arima Genomics Mapping Pipeline (https://github.com/ArimaGenomics/mapping_pipeline) and scaffolded both assemblies with SALSA (Ghurye et al. 2017, 2019).

**Table 1.** Assembly pipeline and software used.

Assembly step	Software and nondefault options	Version	References
Initial assembly			
Filtering PacBio HiFi adapters	HiFiAdapterFilt	Commit 64d1c7b	Sim et al. (2022)
K-mer counting	Meryl (k = 21)	1	https://github.com/marbl/meryl
Estimation of genome size and heterozygosity	GenomeScope	2	Ranallo-Benavidez et al. (2020)
De novo assembly (contiging)	HiFiasm (HiC mode, –primary, output hic.hap1.p_ctg, hic.hap2.p_ctg)	0.16.1-r375	Cheng et al. (2022)
Scaffolding			
Omni-C data alignment	Arima Genomics Mapping Pipeline	Commit 2e74ea4	https://github.com/ArimaGenomics/mapping_pipeline
Arima Genomics Mapping Pipeline (AGMP)	BWA-MEM	0.7.17-r1188	Li (2013)
	samtools	1.11	Danecek et al. (2021)
	filter_five_end.pl (AGMP)	Commit 2e74ea4	https://github.com/ArimaGenomics/mapping_pipeline
	two_read_bam_combiner.pl (AGMP)	Commit 2e74ea4	https://github.com/ArimaGenomics/mapping_pipeline
	picard	2.27.5	https://broadinstitute.github.io/picard/
Omni-C scaffolding	SALSA (-DNASE, -i 20, -p yes)	2	Ghurye et al. (2017), Ghurye et al. (2019)
Omni-C contact map generation			
Short-read alignment	BWA-MEM (-5SP)	0.7.17-r1188	Li (2013)
SAM/BAM processing	samtools	1.11	Danecek et al. (2021)
SAM/BAM filtering	pairtools	0.3.0	Open2C et al. (2024)
Pairs indexing	pairix	0.3.7	Lee at al. (2022)
Matrix generation	cooler	0.8.10	Abdennur and Mirny (2020)
Matrix balancing	hicExplorer (hicCorrectmatrix correct --filterThreshold -2 4)	3.6	Ramírez et al. (2018)
Contact map visualization	HiGlass	2.1.11	Kerpedjiev et al. (2018)
	PretextMap	0.1.4	https://github.com/wtsi-hpag/PretextView
	PretextView	0.1.5	https://github.com/wtsi-hpag/PretextMap
	PretextSnapshot	0.0.3	https://github.com/wtsi-hpag/PretextSnapshot
Manual curation	Rapid curation pipeline (Wellcome Trust Sanger Institute, Genome Reference Informatics Team)	Commit 7acf220c	https://gitlab.com/wtsi-grit/rapid-curation
Genome quality assessment			
Basic assembly metrics	QUAST (--est-ref-size)	5.0.2	Gurevich et al. (2013)
Assembly completeness	BUSCO (-m geno, -l mammalia)	5.0.0	Manni et al. (2021)
	Merqury	2020-01-29	Rhie et al. (2020)
Contamination screening			
Local alignment	BLAST+ (-db nt, -outfmt “6 qseqid staxids bitscore std,” -max_target_seqs 1, -max_hsps 1, -evalue 1e-25)	2.10	Camacho et al. (2009)
General contamination screening	BlobToolKit (PacBIo HiFi Coverage, NCBI Taxa ID = 9696, BUSCODB = mammalia)	2.3.3	Challis et al. (2020)
Mitochondrial assembly			
Mitochondrial genome assembly	MitoHiFi (-r, -p 90, -o 1) Reference: *Puma concolor* (NCBI:NC_016470.1)	2.2	Uliano-Silva et al. (2023)
Mitochondrial genome annotation	MitoFinder (MitoHiFi pipeline parameters)	1.4	Allio et al. (2020)
Synteny analysis			
Pairwise sequence alignment	mummer (nucmer)	3.1	Kurtz et al. (2004)

We manually curated the assemblies for both haplotypes by iteratively generating and analyzing their corresponding Omni-C contact maps. Briefly, to generate the contact maps we aligned the Omni-C data with BWA-MEM (Li 2013) and identified ligation junctions to generate Omni-C pairs (Lee et al. 2022) using pairtools (Open2C et al. 2024). We then generated multiresolution Omni-C matrices with cooler (Abdennur and Mirny 2020) and balanced them with hicExplorer (Ramírez et al. 2018). We used HiGlass (Kerpedjiev et al. 2018) and PretextSuite (https://github.com/wtsi-hpag/PretextView, https://github.com/wtsi-hpag/PretextMap, https://github.com/wtsi-hpag/PretextSnapshot) to visualize the contact maps. We examined the contact maps to identify misassemblies and modified the assemblies using the Rapid Curation pipeline from the Wellcome Trust Sanger Institute’s Genome Reference Informatics Team (https://gitlab.com/wtsi-grit/rapid-curation). We closed some of the gaps using the PacBio HiFi reads and YAGCloser (https://github.com/merlyescalona/yagcloser). We checked for contamination using the BlobToolKit Framework (Challis et al. 2020).

We assigned chromosome names and identified sex chromosomes in our assembly using synteny with the domestic cat (*Felis catus*), Canada lynx (*Lynx canadensis*), and leopard cat (*Prionailurus bengalensis*) genomes. We used nucmer (Kurtz et al. 2004) to align the domestic cat assembly (F.catus_Fca126_mat1.0; NCBI Genome: GCF_018350175.1; includes autosomes, X chromosome, and mitochondrial genome) to our assembly and examined hits longer than 10 kb with greater than 80% sequence identity. We also aligned the leopard cat genome (Fcat_Pben_1.1_paternal_pri; NCBI Genome: GCF_016509475.1; includes autosomes, X chromosome, and mitochondrial genome) to our assembly because the leopard cat shares a karyotype banding pattern with the mountain lion (Wurster-Hill and Centerwall 1982). We identified the Y chromosome in our assembly using the same process with the Canadian lynx Y chromosome (NCBI GenBank: NC_050200.1).

### Genome quality assessment

We generated k-mer counts from the PacBio HiFi reads using meryl (https://github.com/marbl/meryl). We then used the k-mer counts in GenomeScope2.0 (Ranallo-Benavidez et al. 2020) to estimate genome features including genome size, heterozygosity, and repeat content. We ran QUAST (Gurevich et al. 2013) to obtain general contiguity metrics. To evaluate genome quality and functional completeness, we used BUSCO (Manni et al. 2021) with the Mammalia ortholog database (mammalia_odb10), which contains 9,226 genes. We assessed base-level accuracy and k-mer completeness using merqury (Rhie et al. 2020) with the previously generated meryl database. We further estimated genome assembly accuracy using a frameshift analysis of the BUSCO gene set, as described by Korlach et al. (2017). We determined the size of the phased blocks based on the size of the contigs generated by HiFiasm in HiC mode. Following the quality metric nomenclature established by Rhie et al. (2021), for the primary assembly, we calculated the genome quality code *x*.*y*.*P*.*Q*.*C*, where *x* = log10[contig NG50]; *y* = log10[scaffold NG50]; *P* = log10 [phased block NG50]; *Q* = Phred base accuracy QV (quality value); *C* = % genome represented by the first *n* scaffolds, following a karyotype of 2*n* = 38 for *P. concolor* (Challis et al. 2023).

### Mitochondrial genome

We assembled the mitochondrial genome of *P. concolor* from the PacBio HiFi reads using the reference-guided pipeline MitoHiFi (Allio et al. 2020; Uliano-Silva et al. 2023). We used the mitochondrial sequence of a puma (NCBI:NC_016470.1) as the starting sequence. We searched for matches to the resulting mitochondrial assembly sequence in the nuclear genome assembly using BLAST+ (Camacho et al. 2009) and filtered out contigs and scaffolds from the nuclear genome that were shorter than the mitochondrial assembly sequence with greater than 99% sequence identity.

## Results

**Fig. 2.**
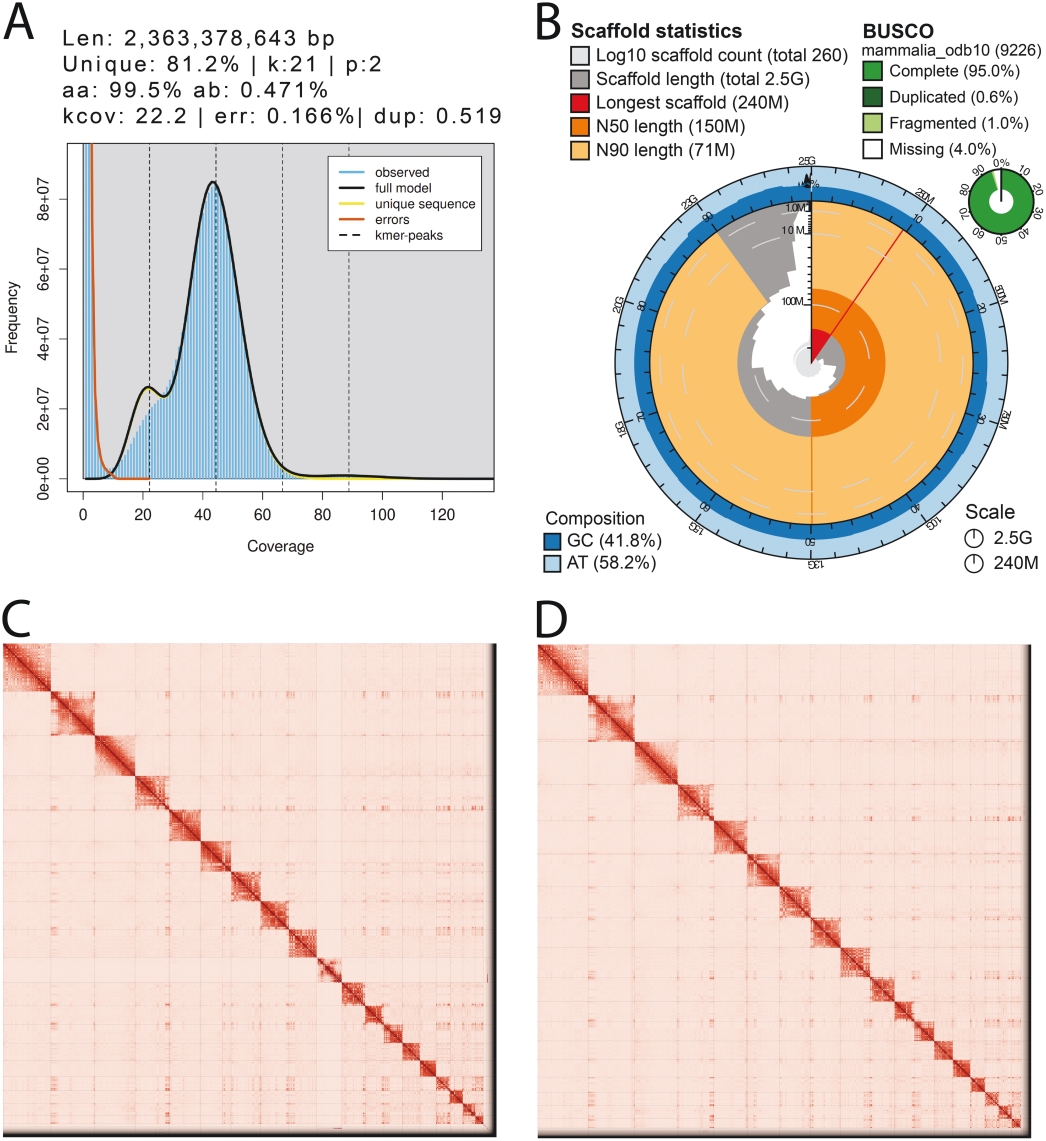
Visualization of assembly metrics. A) K-mer frequencies from the adapter-trimmed PacBio HiFi data used to estimate genome size, sequencing error rate, and heterozygosity. The main peak at ~44-fold coverage corresponds to homozygous regions of the genome, whereas the peak at ~22-fold corresponds to heterozygous regions of the genome. The peak near zero corresponds to probable sequencing errors. B) Snail plot displaying assembly metrics for the primary assembly. C) Omni-C contact map for the primary assembly after manual curation shows the 3D organization of the genome, with darker areas indicating closer proximity. D) Omni-C contact map for the alternate assembly.

The Omni-C library generated 326.75 million read pairs and the PacBio HiFi SMRTbell library generated 6.43 million HiFi reads. The PacBio HiFi reads yielded ~46-fold genome coverage and had an N50 read length of 17,027 bp, a minimum read length of 66 bp, a mean read length of 16,913 bp, and a maximum read length of 58,906 bp (see Supplementary Fig. S1 for read length distribution). Based on the PacBio HiFi data, Genomescope 2.0 estimated a genome size of 2.36 Gb, a 0.166% sequencing error rate, and 0.471% heterozygosity. The k-mer spectrum shows a bimodal distribution with a major coverage peak at ~44-fold coverage and a minor coverage peak ~22-fold coverage (Fig. 2A).

The final genome assembly (mPumCon1) consists of two phased haplotypes. The primary haplotype consists of autosomes, sex chromosomes, and the mitochondrion; whereas the alternate haplotype contains only autosomes. Both assemblies are similar in size but not equal to the estimated genome size from GenomeScope2.0, as has been observed in other taxa (see Pflug et al. 2020, for example). The primary assembly (mPumCon1.0.hap1) consists of 258 scaffolds spanning 2.50 Gb with a contig N50 of 42.3 Mb, a scaffold N50 of 149.8 Mb, the largest contig size of 110.9 Mb, and the largest scaffold size of 242.3 Mb (Fig. 2B). The alternate assembly (mPumCon1.0.hap2) consists of 207 scaffolds spanning 2.37 Gb with a contig N50 of 65.8 Mb, a scaffold N50 of 149.5 Mb, the largest contig size of 146.9 Mb, and the largest scaffold size of 242.4 Mb.

The primary assembly has a BUSCO completeness score for the Mammalia gene set of 95.0%, a base pair QV of 63.01, a k-mer completeness of 97.4%, and a frameshift indel QV of 43.91. The alternate assembly has a BUSCO completeness score for the Mammalia gene set of 93.8%, a base pair QV of 62.68, a k-mer completeness of 93.48%, and a frameshift indel QV of 46.76.

During manual curation, we made a total of 121 joins (67 on the primary assembly and 54 on the alternate assembly) and 20 breaks (10 per assembly) based on signals in the Omni-C contact maps. We filtered out a single contig corresponding to mitochondrial contamination. The Omni-C contact maps for both assemblies show chromosome-level assemblies (Fig. 2C and D). Assembly statistics are reported in Table 2 and represented graphically in Fig. 2B. We have deposited the genome assembly at NCBI GenBank (see Table 2 and Data Availability for details).

**Table 2.** Assembly statistics and data availability.

Bio Projects and Vouchers	CCGP NCBI BioProject			PRJNA720569			
	Genera NCBI BioProject			PRJNA765839			
	Species NCBI BioProject			PRJNA777212			
	NCBI BioSample			SAMN30567381			
	Specimen identification			L17-30			
	NCBI Genome accessions			**Primary**		**Alternate**	
	Assembly accession				JAQJVZ000000000	JAQJWA000000000	
	Genome sequences			GCA_028749985.4		GCA_028749965.4	
Genome Sequence	PacBio HiFi reads		Run	PACBIO_SMRT (Sequel II/IIe):			
				6.4M spots, 108.8G bases, 72.3G bytes			
			Accession	SRX21253672			
	Omni-C Illumina reads		Run	ILLUMINA (Illumina NovaSeq 6000):			
				226.8M spots, 98.7G bases, 34.2G bytes			
			Accession	SRX21253673, SRX21253674			
Genome Assembly Quality Metrics	Assembly identifier (Quality code^a^)				7.8.P7.Q63.C98		
	HiFi Read coverage^b^			46.03X			
				**Primary**		**Alternate**	
	Number of contigs			403		291	
	Contig N50 (bp)			42,297,498		65,828,543	
	Contig NG50^b^			50,506,965		65,828,543	
	Longest Contigs			110,887,988		146,946,459	
	Number of scaffolds			258		207	
	Scaffold N50			149,756,039		149,508,014	
	Scaffold NG50^b^			149,756,039		149,508,014	
	Largest scaffold			242,276,767		242,393,222	
	Size of final assembly			2,501,085,587		2,368,682,789	
	Phased block NG50^b^				50,506,965		65,828,543
	Gaps per Gbp (# Gaps)			58 (145)		35 (84)	
	Indel QV (Frame shift)			43.91		46.76	
	Base pair QV (Full assembly = 62.86)				63.01	62.68	
	k-mer completeness (Full assembly = 99.7174)				97.4057	93.4802	
	BUSCO^c^ completeness (mammalia) n = 9,226		**C**	**S**	**D**	**F**	**M**
		P^d^	95.01%	94.44%	0.57%	1.03%	3.96%
		A^d^	93.82%	93.27%	0.55%	1.05%	5.13%
	Organelles		1 mitochondrial sequence			CM053820.1	

^a^Assembly quality code *x*.*y*.*P*.*Q*.*C* derived notation from the study by Rhie et al. (2021). *x* = log10[contig NG50]; *y* = log10[scaffold NG50]; *P* = log10 [phased block NG50]; *Q* = Phred base accuracy QV; *C* = % genome represented by the first *n* scaffolds, following a known karyotype for *P. concolor* of 2*n* = 38 (Challis et al. 2023). Quality code metrics were calculated from the primary assembly (mPumCon1.0.hap1).

^b^Read coverage and NGx statistics have been calculated based on the estimated genome size of 2.36 Gb.

^c^BUSCO scores. Complete BUSCO (C). Complete and single-copy BUSCO (S). Complete and duplicated BUSCO (D). Fragmented BUSCO (F). Missing BUSCO (M).

^d^(P)rimary and (A)lternate assembly values.

We assembled a complete mitochondrial genome for *P. concolor* with a final size of 17,153 bp; a base composition of *A* = 32.851%, *C* = 25.995%, *G* = 13.858%, *T* = 27.296%; and consisting of 2 rRNAs, 22 unique transfer RNAs, and 13 protein coding genes.

## Discussion

We generated a high-quality, chromosome-level genome assembly for a mountain lion from southern California. Our new genome assembly is a critical resource for ongoing research using population and landscape genomic analyses to aid in the management of mountain lion populations. High-quality genome assemblies are a foundational component of population assessments of genetic diversity, deleterious load, and inbreeding and enable the identification of barriers to gene flow in fragmented habitats. The knowledge gained from these analyses can be used to parameterize models that simulate population dynamics across space and through time to better understand the consequences of different management scenarios (Benson et al. 2016, 2019).

This assembly has improved contiguity over our previously published assembly for a mountain lion from the central coast of California, which was assembled to near-chromosome level (NCBI RefSeq accession GCF_003327715.1; Saremi et al. 2019). The scaffold N50 increased from 100.5 Mb to 149.8 Mb and the number of scaffolds decreased from 2,174 to 258. The increased contiguity of the new assembly will enable improved analysis of linked markers, such as identifying genomic tracts that are shared identical-by-descent (IBD) between individuals. Accurately identifying IBD tracts allows for examination of spatial connectivity over time, which is critical to understanding species living in increasingly fragmented landscapes. Additionally, our new chromosome-level assembly can be analyzed with the more than a dozen other chromosome-level felid assemblies to identify variation underlying important adaptations in this diverse family.

## Supplementary material

Supplementary material is available at *Journal of Heredity* online.

esae063_suppl_Supplementary_Figures_S1

## Data Availability

Data generated for this study are available under NCBI BioProject PRJNA777212. Raw sequencing data for sample L17-30 (NCBI BioSample SAMN30567381) are deposited in the NCBI Short Read Archive (SRA) under experiment accessions SRX21253672 (PacBio HiFi sequencing data) and SRX21253673-SRX21253674 (Omni-C Illumina sequencing data). GenBank accessions for the assemblies are GCA_028749985.4 (primary) and GCA_028749965.4 (alternate), with nucleotide sequences under accessions JAQJVZ000000000 and JAQJWA000000000, respectively. GenBank accession for the mitochondrial assembly is CM053820.1. Assembly workflow is available at www.github.com/ccgproject/ccgp_assembly.
