# Supplementary material for: A chromosome-level genome assembly of the mountain lion, Puma concolor
Source: J Hered. 2024 Nov 7;116(4):479–87. doi: 10.1093/jhered/esae063 (PMC12277564; doi:10.1093/jhered/esae063)

**Supplementary Figure S1.** Read length distribution of PacBio HiFi reads. The red line indicates the average read length.


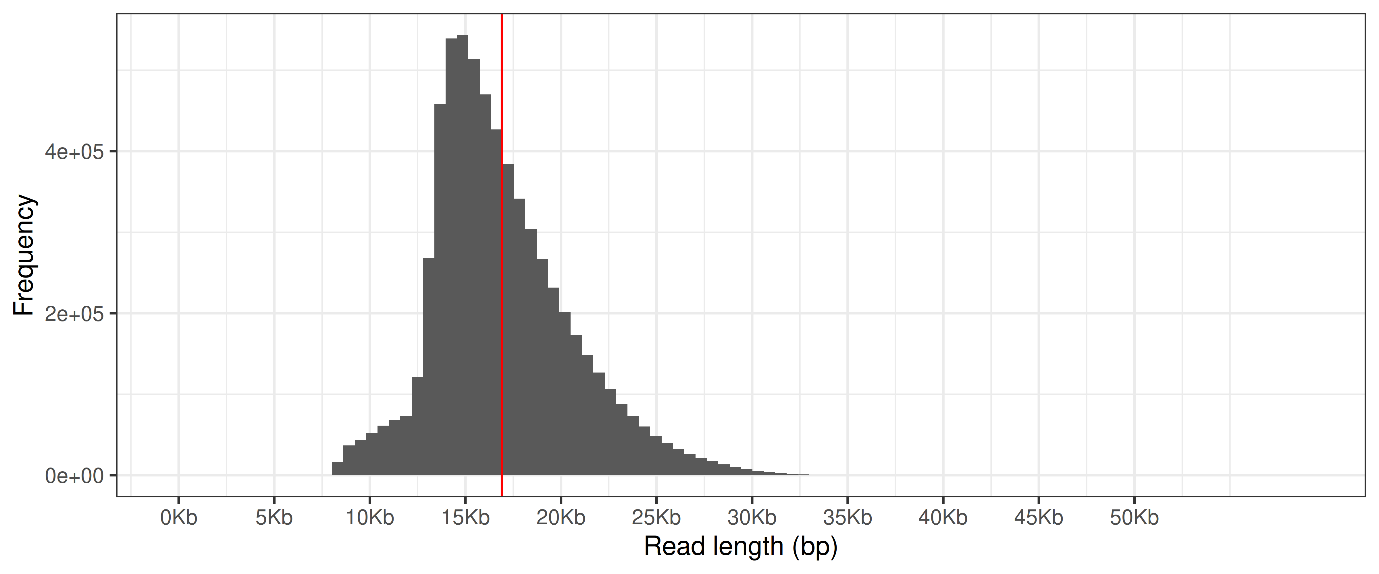

Supplement: esae063_suppl_Supplementary_Figures_S1 [file esae063_suppl_supplementary_figures_s1.docx]
